# Doravirine versus dolutegravir-based regimen in antiretroviral treatment-naive people living with HIV-1 (ANRS0392s ELDORADO): protocol for an international, open-label, randomised, non-inferiority, phase III trial

**DOI:** 10.1136/bmjopen-2025-110560

**Published:** 2026-02-05

**Authors:** Anthony L’hostellier, Charles Kouanfack, Corine Chazallon, Sandra Wagner-Cardoso, Serge Paul Eholie, Neiva Banze, Guttiga Halue, Jacqueline Capeau, Constance Delaugerre, Raoul Moh, Fabrice Bonnet, Liliane Mfeukeu Kuate, Antoine Jaquet, Hugo Perazzo, Charlotte Bernard, Jean-Philippe Bastard, Lauriane Goldwirt, Paul Vilquin, Pedroso Pedro Nhassengo, Margot Lavalée, Nicolas Minvielle, P J Dodd, Olivier Marcy, Jean-Michel Molina, Beatriz Grinsztejn, Pierre O Sellier

**Affiliations:** 1 National Institute for Health and Medical Research (INSERM) UMR 1219, Research Institute for Sustainable Development (IRD) EMR 271, Bordeaux Population Health Research Center, University of Bordeaux, Bordeaux, France; 2 Faculty of Medicine and Pharmaceutical Sciences, Université de Dschang, Yaounde, Cameroon; 3 STDs/AIDS Clinical Research Laboratory, Oswaldo Cruz Foundation, National Institute of Infectology (INI), Rio de Janeiro, Brazil; 4 Department of Infectious and Tropical Diseases, CHU de Treichville, Abidjan, Côte d’Ivoire; 5 Instituto Nacional de Saúde, Maputo, Mozambique; 6 Internal Medicine Department, Department of Medical, Phayao Hospital, Mueang Phayao District, Thailand; 7 Inserm UMR_S938, CRSA, ICAN, Sorbonne Universite, Paris, France; 8 Virology Department, Assistance Publique - Hopitaux de Paris, Paris, France; 9 Programme PAC-CI, Abidjan, Côte d’Ivoire; 10 Hôpital St-André, Internal Medicine and Infectious Diseases Department, CHU de Bordeaux, Bordeaux, France; 11 Universite de Yaounde I Faculte de Medecine et des Sciences Biomedicales, Yaounde, Cameroon; 12 Department of Biochemistry - Pharmacology, Assistance Publique - Hopitaux de Paris, Paris, France; 13 INSERM UMR 955 and UPEC, Faculty of Health, Créteil, France; 14 Pharmacology Department, Saint-Louis Hospital, GHU AP-HP Nord Université Paris Cité, Paris, France; 15 Delegação Provincial da Cidade de Maputo, Instituto Nacional de Saúde, Maputo, Mozambique; 16 Agence Nationale de Recherches sur le sida et les hépatites virales ANRS|Maladies infectieuses émergentes, Paris, France; 17 School of Health and Related Research, University of Sheffield, Sheffield, UK; 18 Department of Infectious Diseases, AP-HP Saint-Louis and Lariboisière Hospitals, Paris, France; 19 Paris Cité University, Paris, France

**Keywords:** INFECTIOUS DISEASES, Clinical trials, Protocols & guidelines, HIV & AIDS, Public health

## Abstract

**Introduction:**

Increasing evidence suggests that dolutegravir (DTG), endorsed by the WHO since 2018 for first-line antiretroviral therapy (ART), is associated with significant weight gain and potentially also with cardiometabolic disorders. In an effort to expand therapeutic options for people living with HIV (PLHIV), the EvaLuating the non-inferiority of DORAvirine vs DOlutegravir trial aims to compare the virologic efficacy of doravirine (DOR) and DTG-based regimens and to assess their safety, including a focus on cardiometabolic effects.

**Methods and analysis:**

This is an international, phase III, multicentre, open-label, non-inferiority, randomised trial that will enrol 610 ART-naïve PLHIV (HIV RNA≥1000 copies/mL at screening) across six countries (Brazil, Cameroon, France, Côte d’Ivoire, Mozambique and Thailand) spanning four continents. Key inclusion criteria include age ≥18 years, confirmed HIV-1 infection with plasma RNA levels ≥1000 copies/mL, indication for ART initiation and no prior ART exposure. Participants will be randomised in a 1:1 ratio to receive either DOR 100 mg once daily in combination with tenofovir disoproxil fumarate (TDF) (300 mg daily) plus lamivudine (3TC) (300 mg daily) or DTG (50 mg daily) in combination with TDF (300 mg once daily) plus either emtricitabine (FTC) (200 mg daily) or 3TC (300 mg daily). Randomisation will be stratified by screening HIV-1 RNA load (≤100 000 or >100 000 copies/mL) and by country. The primary outcome is virological efficacy, defined as the proportion of participants achieving HIV-1 RNA <50 copies/mL at week 48 on the assigned treatment (FDA Snapshot algorithm). Secondary outcomes include cardiometabolic safety endpoints (ie, weight gain, insulin resistance, hypertension, diabetes, waist and hip circumferences, waist-to-hip ratio, fasting glycaemia, insulin and fasting serum lipids), along with mental health, quality of life, virological and immunological parameters. Final data collection is expected by July 2028.

**Ethics and dissemination:**

Primary outcome results (week 48) are expected in early 2028. The project was submitted to and approved by national ethics committees and pharmaceutical regulatory authorities in all participating countries: Brazil (CEP INI FIOCRUZ (21.040-900)/CEP HGNI (26.030-380)); Cameroon (CNERSH (2024/09/1717/CE/CNERSH/SP)/Ministry of Public Health (D30-1464/AAR/MINSANTE/SG/DROS/CRC); Côte d'Ivoire: (CNESVS (0018224/MSHPCMU/CNESVS-km)/AIRP (1329/AIRP/DISMP/Om/kbaag); France (CTIS CPP/ANSM (2023-508626-10-00)); Mozambique (CNBS (20/CNBS/25)/ANARME (4635/380/ANARME)); Thailand: (IHRP (08/1944)/Thai FDA: ongoing on 19 January 2026). The trial received authorisation from the French National Commission for Data Protection and Liberties (CNIL) under approval number 924 302. Written informed consent is obtained from all participants prior to any study-specific procedures and trial enrolment, in accordance with the Declaration of Helsinki and applicable national regulations. Study findings will be disseminated through publication in peer-reviewed journals and presentations at national and international scientific conferences. Results will also be communicated to policymakers, healthcare professionals, community stakeholders and study participants through appropriate dissemination activities, including policy briefs, stakeholder meetings and lay summaries on dedicated and easily accessible platforms.

**Trial registration numbers:**

NCT06203132; EU-CT, 2023-508626-10-00.

**STRENGTHS AND LIMITATIONS OF THIS STUDY**

- Participants will be recruited from ethnically diverse populations across Latin America, sub-Saharan Africa, Europe and Asia; such diversity is underrepresented in conventional phase III randomised clinical trials but is critical to extrapolate the results, both in terms of effectiveness and safety endpoints.
- The use of tenofovir disoproxil fumarate (TDF), rather than tenofovir alafenamide fumarate (TAF), which may also contribute to weight gain, in both arms, will allow for a clearer assessment of the specific impact of the third agent (doravirine or dolutegravir) on weight gain.
- Selecting a therapeutic regimen based on TDF instead of TAF allows evaluation of the WHO-recommended regimen among antiretroviral therapy-naïve people living with HIV.
- 2 years of follow-up may be too short to assess possible cardiometabolic events.

## Introduction

### Background and rationale

 From 39 million people living with HIV (PLHIV) worldwide in 2023, only 29.8 million accessed antiretroviral therapy (ART).^1^ International guidelines recommend using a second generation integrase strand transfer inhibitor (INSTI), dolutegravir (DTG) or bictegravir, in combination with a nucleoside/nucleotide reverse transcriptase inhibitor (NRTI) backbone as preferred first-line regimen,^2 3^ and the WHO recommends using DTG in combination with tenofovir disoproxil fumarate (TDF) and XTC (3TC or FTC) as the preferred NRTI for first-line regimen since 2018.^3^ As of June 2021, 110 low-income and middle-income countries had transitioned to DTG and an estimated 22 million PLHIV received DTG-based ART.^4^

Globally, recent programme and trial data in adults following ART initiation suggest that INSTI-based ARTs are associated with higher weight gain compared with other antiretroviral-based regimens.^5^,^7^ Growing evidence indicates that DTG-based regimens have a significant impact on body weight,^4^ which could be higher compared with other INSTIs.^56 8^,^10^ Women, black people and TAF use are associated with an increased risk of greater weight gain.^811^,^13^ In the ANRS 12313 NAMSAL trial, conducted in Cameroon, the weight gain at week 48 was greater in the DTG group (median+5.0 kg) than in the efavirenz (EFV) group (+3.0 kg), p<0.001.^6^ The incidence of obesity over the first 48 weeks was 12.3% in the DTG group vs 5.4% in the EFV group. At week 96, the incidence of obesity was 22% in the DTG group vs 16% in the EFV group, p=0.043. At week 196, the median weight gain was +7.0 kg in the DTG group, whereas it was +5.0 in the EFV group, p<0.001.^14^ In the ADVANCE trial, conducted in South Africa, weight increase at week 48 was greatest in the TAF-based group and among female participants (mean+6.4 kg in the TAF/FTC/DTG group, +3.2 kg in the TDF/FTC/DTG group and +1.7 kg in the TDF/FTC/EFV group). At week 96, the mean weight gain was +7.1 kg in the TAF/FTC/DTG group, +4.3 kg in the TDF/FTC/DTG group and +2.3 kg in the TDF/FTC/EFV group, and it was greater among female than male participants. Risk factors for weight gain in individuals initiating ART were CD4 counts <200 cells, HIV RNA levels >100 000 copies/mL, normal body mass index (BMI) at initiation, female sex at birth, ethnicity, using bictegravir, DTG or rilpivirine rather than EFV and using TAF rather than TDF.^8^ Results from clinical trials have been confirmed by data from observational cohorts showing significant weight gain in PLWIH switching to a DTG-based regimen compared with non-switchers.^15^,^17^

Beyond weight gain, some recent data reported an association between INSTI use and the onset of hypertension, and possibly also with type 2 diabetes mellitus.^18^,^20^ However, in addition to conflicting results from observational data, uncertainties remain regarding the mechanistic pathways driving those potential effects.^21 22^ Aside from clinical disorders, mental health disorders, including anxiety, depression and sleep disorders, have been associated in many observational studies with the use of INSTI and particularly DTG.^23 24^ Therefore, addressing the impact of DTG on safety outcomes deserves additional data through well-conducted experimental studies.

Doravirine (DOR), a potent NNRTI, is seen as a possible alternative to DTG-based regimens. In combination with TAF or TDF and FTC or 3TC, DOR is recommended as first-line ART in the 2024 European AIDS Clinical Society Guidelines and as an alternative to INSTI in the 2024 IAS-USA Guidelines in HIV-1 infected subjects.^2 25^

Evidence from the literature provides indirect support for the non-inferiority of DOR-based regimens compared with INSTI-based regimens in treatment-naïve PLHIV.^26^,^28^ In the DRIVE-AHEAD phase 3, double-blind trial comparing DOR to EFV in ART-naive adults, non-inferiority was demonstrated at 48 weeks.^24^ DOR has also been compared with ritonavir-boosted darunavir,^11^ with non-inferiority confirmed at both week 48 and week 96. Importantly, weight gain in the DOR arms was quite low: median weight gains at weeks 48, 96, 148 and 192 were +1.4 kg, +0.9 kg, +1.6 kg and +1.1 kg in Drive-Forward, respectively; and +2.0 kg, +1.9 kg, +1.9 kg and +2 kg at the same weeks in Drive-Ahead. Therefore, given that: (1) treatment with DTG has been associated with weight gain, particularly in female subjects, and in subjects from sub-Saharan origin^6 7 29^ and its concerning long-term consequences such as increased weight gain regarding cardiovascular risk, hypertension and diabetes^1630^,^32^; (2) DOR has been associated with moderate weight gain comparable to that observed with other non-INSTI molecules^28^ and (3) there is no ongoing study comparing DOR to DTG with the same NRTI backbone of TDF/XTC in ART naïve; a first-line trial assessing the non-inferiority of DOR and better safety profile as compared with DTG in a diverse population is likely to have a significant impact in terms of public health by contributing to change international guidelines.

## Methods and analysis

### Study design

This study, ongoing in over 19 investigational sites in 6 countries (Brazil, Cameroon, France, Côte d’Ivoire, Mozambique and Thailand), is a multicentre, open-label, randomised, active-controlled, non-inferiority trial of DOR 100 mg once daily in combination with TDF (300 mg daily) plus 3TC (300 mg daily) in a single tablet regimen (STR) compared with DTG (50 mg daily) in combination with TDF (300 mg daily) plus FTC (200 mg daily) or 3TC (300 mg daily) regimen (2 combined tablets in France and Brazil; STR in countries with access to the DTG/TDF/3TC fixed dose combination) in ART-naïve PLHIV. A double-blind design was not feasible due to differing pill formulations and local STR availability.

### Study population and recruitment

To be eligible for participation, individuals must be aged ≥18 years, HIV-1 positive, have plasma HIV-1 RNA levels ≥1000 copies/mL, meet criteria for HIV treatment initiation and be ART-naïve. The rationales of using the cut-off of baseline RNA levels of ≥1000 copies/mL were: (1) this cut-off was previously used in the two large RCTs comparing DOR to ritonavir-boosted darunavir,^11^ and to EFV,^24^ and in the NAMSAL trial, comparing DTG to EFV.^6^ In the ADVANCE trial, the cut-off was 500 copies/mL. (2) Sites participating in this international trial used the cut-off of 1000 copies/mL in routine. Female subjects must have a negative urinary pregnancy test and agree to use contraceptive methods. Subjects with chronic viral hepatitis (B and/or C) will be included, provided they fulfil all entry criteria, have stable liver function tests and no significant impairment of hepatic synthetic function. Eligibility criteria are summarised in box 1. All participants must understand the study procedures and voluntarily agree to participate by giving written informed consent (online supplemental file 1). Initial HIV-1 treatment-naïve status will be assessed in accordance with the national guidelines in each participating country. HIV diagnosis will follow the standard diagnostic algorithm, including an initial rapid diagnostic test followed by a discriminatory confirmatory assay performed during screening visit to exclude HIV-2 infection or HIV-1/HIV-2 coinfection. Treatment-naïve status will be determined based on a detailed clinical history, review of available medical records and confirmation of no prior exposure to ART. This approach reflects routine clinical practice in the study settings and ensures reliable identification of ART-naïve participants.

Box 1 **Overview of eligibility criteria**

**Inclusion criteria**

- Age ≥18 years.
- HIV-1 positive with plasma HIV-1 RNA≥1000 copies/mL.
- HIV treatment indication according to national guidelines.
- ART-naïve.

Note: Subjects having received oral pre-exposure prophylaxis (PrEP) or postexposure prophylaxis (PEP) are eligible, if treatment ended more than 3 months before HIV-1 diagnosis and a negative HIV-1 test is documented in-between.

- For female subjects of childbearing potential, a negative urinary test for pregnancy and acceptance to use contraceptive methods.
- For patients in France, being affiliated to a social security programme.

**Exclusion criteria**

- Ongoing tuberculosis disease.
- Any history, condition, therapy, laboratory abnormality or other circumstances that might interfere with the subject’s participation for the full duration of the study.
- Infection or coinfection with HIV-2.
- Previous cabotegravir or dapivirine PrEP.
- Previous oral PrEP or PEP within the past 3 months.
- Documented or known HIV resistance to study drugs.
- Within 30 days prior to randomisation:
  - Aspartate aminotransferase (AST) (serum glutamic-oxaloacetic transaminase, SGOT) and alanine aminotransferase (ALT) (serum glutamic-pyruvic transamine, SGPT) >4.0×upper limit of normal.
  - Estimated glomerular filtration rate <60 mL/min/1.73 m² (using Chronic Kidney Disease Epidemiology Collaboration (CKD-EPI) equation).
- Participation in a study with an investigational compound/device within 30 days prior to randomisation.
- Systemic immunosuppressive therapy or immune modulators use within 30 days prior to randomisation.
- Pregnant, breastfeeding or expecting to conceive during the study.
- Under guardianship or deprived of freedom.

### Medical intervention

610 participants will be randomised into one of the two following arms:

- DOR arm: participants (n=305) will receive DOR+TDF+3 TC for 96 weeks.
- DTG arm: participants (n=305) will receive DTG+TDF+XTC for 96 weeks.

In the DOR arm, participants will receive the following medications: MK-1439A once daily: DOR (100 mg daily) plus TDF (300 mg daily) plus 3TC (300 mg daily) in a STR. In the DTG arm, participants will receive the following medications, in accordance with national treatment guidelines in participating countries: DTG (50 mg daily) in combination with TDF (300 mg daily) plus FTC (200 mg daily) or 3TC (300 mg daily)—in two tablets in France and Brazil, and STR in countries with access to the DTG/TDF/3TC fixed dose combination. For all participants with a CD4+T cell count below the national threshold in need to initiate *Pneumocystis carinii* pneumonia (PCP) prophylaxis, trimethoprim-sulfamethoxazole (cotrimoxazole) or an alternative option in case of intolerance to cotrimoxazole will be suggested.

In high tuberculosis (TB) incidence countries where TB preventive therapy (TPT) is recommended for PLHIV, participants in the DTG arm will be offered the nationally recommended TPT regimen. This may include 6 months of daily isoniazid (6H) or, where available, rifapentine-based regimens such as 3HP (weekly rifapentine and isoniazid for 12 weeks). For rifapentine-based regimens, DTG will be administered at the standard once-daily dose, in line with WHO, Brazil, France, Cameroon, Côte d’Ivoire, Mozambique and Thailand guidelines, all of which do not recommend DTG dose adjustment when co-administered with 3HP. In the DOR arm, only 6 hour will be allowed, due to drug–drug interactions between DOR and rifamycins (rifampicin and rifapentine); no other TPT regimens will be permitted in this arm.

If TB occurs during the trial in the DOR arm, DOR should be discontinued and the participant should be switched to DTG twice a day (BID) according to the physician’s decision, drug availability and national guidelines. If TB occurs in the DTG arm, dosage should be doubled (BID). Participants in whom TB occurs during the trial will be followed until week 96 but will be considered as a ‘strategic failure’ and will fall in the ‘no data’ category.

### Primary and secondary outcomes

#### Primary virologic outcome

The primary virologic outcome is the proportion of participants achieving virologic success, defined as HIV-1 RNA levels <50 copies/mL at week 48 under the allocated treatment, assessed using the Food and Drug Administration (FDA) Snapshot algorithm within a window period of 42–54 weeks.

#### Secondary virologic outcomes

1. Proportion of participants achieving HIV-1 RNA <50 copies/mL at week 96 (FDA Snapshot, window 90–102 weeks).
2. Proportion of confirmed virologic failure through week 48 and 96, defined as (1) HIV-1 RNA≥200 copies/mL after initial suppression (<50 copies/mL) at any point or (2) non-response (confirmed HIV-1 RNA≥200 copies/mL at week 24 or 36, or ≥50 copies/mL at weeks 48, 72 or 96, or equivalent unscheduled visits).
3. Frequency of HIV-1 drug resistance mutations among participants with virologic failure, interpreted using the latest ANRS and Stanford algorithms.
4. Proportion of participants achieving HIV-1 RNA <200 copies/mL and <1000 copies/mL at weeks 48 and 96.
5. Frequency of reverse transcriptase (RT) and integrase mutations at baseline and impact on virologic response at weeks 48 and 96.

#### Key safety and metabolic outcomes

1. Obesity at weeks 48 and 96, defined as BMI ≥30 kg/m² (Caucasian/African population) or ≥27.5 kg/m² (Asian population).
2. Newly insulin resistance is defined as HOMA (= glucose [mmol/L] × insulin [mIU/L]) ÷ 22.5) ≥2 at weeks 48 and 96.
3. Newly detected hypertension is defined as new antihypertensive treatment and/or diastolic BP >90 mm Hg or systolic BP >140 mm Hg, during a visit and confirmed during a subsequent visit >15 days after.

#### Additional metabolic and clinical safety outcomes

1. Occurrence of combined overweight/obesity (BMI≥25 kg/m² (for Caucasian/African population) or ≥23 kg/m² (for Asian population) at weeks 48 and 96.
2. Proportion of subjects with ≥10% absolute weight gain from baseline at weeks 48 and 96.
3. Change from baseline in absolute weight at weeks 48 and 96.
4. Proportion with newly detected diabetes at Weeks 48 and 96, defined as either being prescribed new medication for diabetes mellitus and/or having a fasting glycaemia ≥1.26 g/L during a visit and confirmed during a subsequent visit >15 days after and/or in a patient with classic symptoms of hyperglycaemia or hyperglycaemic crisis, a random plasma glucose ≥200 mg/dL (11.1 mmol/L).
5. Any adverse event of any grade and those graded 3–4 at weeks 48 and 96.
6. Change from baseline in waist and hip circumferences and waist-to-hip ratio at weeks 48 and 96.
7. Change from baseline in fasting glycaemia and insulin at weeks 48 and 96.
8. Change from baseline in fasting serum lipids profile (total cholesterol, high-density lipoprotein (HDL), low-density lipoprotein (LDL) and triglycerides) at weeks 48 and 96.
9. Change from baseline in estimated glomerular filtration rate, calculated using CKD-EPI calculation) at weeks 48 and 96.
10. Change from baseline in cardiovascular parameters (blood pressure profile, electrocardiographic and echocardiographic damages) at weeks 48 and 96 measured through an ECG, a transthoracic echocardiography and an Ambulatory Blood Pressure Monitoring (ABPM), a 24-hour blood pressure Holter.
11. Change from baseline in hepatic parameters using vibration controlled transient elastography (VCTE) by Fibroscan (EchoSens, Paris, France).
  - Liver steatosis is defined by VCTE-controlled attenuation parameter (CAP) ≥263 dB/m^2^, and clinically significant liver fibrosis (CSF) and cirrhosis defined by VCTE-liver stiffness measurement (LSM)≥8.0 kPa and LSM≥12.5 kPa, respectively.
  - Metabolic dysfunction-associated liver disease (MASLD) is defined by the presence of liver steatosis with at least one cardiometabolic risk factor (overweight/obesity; pre-diabetes; hypertension; hypertriglyceridaemia or low-HDL; see section 9.7 for detailed definitions), and metabolic dysfunction-associated steatohepatitis (MASH) defined by the presence of at least CSF in people with MASLD.
12. Changes from baseline in VCTE, FIB-4 and FAST scores at weeks 48 and 96 and presence at baseline or occurrence of:
  - CSF defined as FIB-4≥2.67.
  - FAST score >0.67 (high probability of MASH).
13. Change from baseline in EuroQol five-dimensions three-levels (EQ-5D-3L), HIV Treatment Satisfaction Questionnaire (HIVTSQ), Depression, Anxiety and Stress Scale - 21 (DASS-21), Pittsburgh Sleep Quality Index (PSQI), World Health Organization Quality of Life - HIV-BREF (WHOQOL HIV-BREF) scores at weeks 24, 48 and 96.
14. Proportion of subjects with AIDS (defined as having CDC stage 3/C or WHO stage 4), TB, IRIS or death at weeks 48 and 96.

#### Other outcomes

1. ARV trough levels (DOR, DTG, M9) at weeks 4, 24, 48 and 96.
2. Changes in CD4/CD8 cell counts, percentages and CD4/CD8 ratio at weeks 48 and 96.
3. ART adherence through pill count (<95% considered suboptimal), recall methods and TFV-DP levels in Dried Blood Spots (DBS at weeks 48 and 96.
4. Pharmacogenetic markers: CYP3A5/4 allelic variants, their impact on drug pharmacokinetics, virologic outcomes and safety.
5. Baseline assessment of UGT1A1 polymorphisms.
6. Health economic evaluations including health impact (disability-adjusted life-years (DALYs), quality-adjusted life-years (QALYs)), economic costs, cost-effectiveness and budget impact.

#### Metabolic substudy outcomes (80 women (40 per arm))

1. Change from baseline in truncal fat distribution (using DEXA-scanner assessments) at weeks 48 and 96.
2. Changes from baseline in serum adipose (adiponectin, leptin) and immune activation markers (sCD14, sCD163) at weeks 48 and 96.
3. Changes from baseline in fat tissue pathology, gene expression and global transcriptome analysis (RT-PCR and RNAseq) at week 48.

### Study procedures

**Figure 1:**
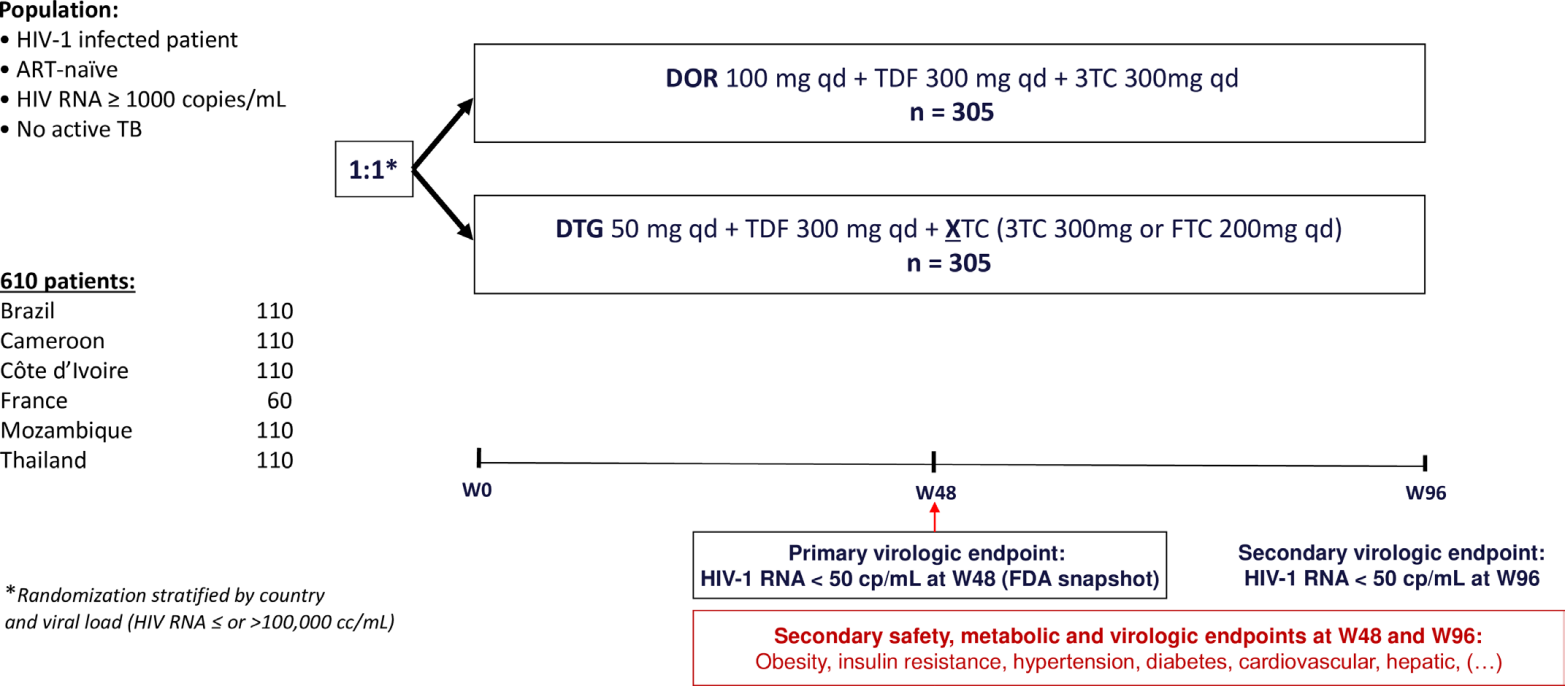
ANRS0392s ELDORADO trial diagram. ELDORADO, EvaLuating the non-inferiority of DORAvirine vs DOlutegravir; ART, antiretroviral therapy; TB, tuberculosis; DOR, doravirine; DTG, dolutegravir; TDF, tenofovir disoproxil fumarate; FTC, emtricitabine; FDA, food and drug administration.

The trial design is described in figure 1.

ART-naïve participants (with HIV-1 RNA≥1000 copies/mL) newly registered in participating trial sites will be offered to participate in the study starting from February 2025 and ending in July 2028. The planned recruitment period is 12 months in each country. We expect to have finished recruitment (N=610) by the end of 2026. Informed consent will be obtained before any study procedure under the protocol and at the latest before pre-inclusion (screening) visit. In addition to oral explanations, a written information sheet will be systematically provided. The screening window is up to 30 days prior to HIV treatment initiation and will include the following procedures: obtaining written consent, verification of eligibility criteria, clinical evaluation: demographic information, history of HIV-associated conditions and other relevant medical history, WHO-recommended four questions for TB screening, complete physical exam, current medical conditions and medications, systematic chest radiography to rule out TB disease, local laboratory assessment: discriminant HIV test, complete blood count (CBC), AST, ALT, creatinine blood levels and creatinine clearance calculation, CD4 and CD8 cell count and percentages, CD4/CD8 ratio, plasma HIV-1 RNA±urine pregnancy tests. The corresponding electronic case report forms, including scheduling of the inclusion visit, will be completed as promptly as possible.

### Randomisation

For participants who meet all inclusion criteria and none of the exclusion criteria, once the investigator or physician has obtained and verified all required results, the participant will be randomised into one of the two trial arms. Randomisation will be stratified by the levels of the HIV-1 RNA load (> or < 100 000 c/mL) and by country. Randomisation will be stratified by baseline HIV-1 RNA level (≤100 000 vs >100 000 copies/mL) because baseline viral load is a well-established prognostic factor for virological response to ART. Higher baseline HIV-1 RNA levels have been associated with delayed viral suppression and an increased risk of early virological failure. The threshold of 100 000 copies/mL is widely used in clinical trials and international guidelines as a clinically meaningful cut-off to distinguish patient populations with differing virological risk profiles. Stratifying randomisation by this variable ensures comparability between treatment arms, minimises potential confounding and strengthens the internal validity of the study outcomes. Moreover, randomisation stratified by baseline HIV-1 RNA level (≤100 000 vs >100 000 copies/mL) has been used previously in the two large RCTs comparing DOR to ritonavir-boosted darunavir,^11^ and to EFV,^24^ as in NAMSAL and ADVANCE trials, comparing DTG to EFV.^6 28^ Randomisation will also be stratified by country as data concerning virological success are scarce, mainly for DOR, towards non-B HIV-1 strains; moreover, weight gain in PLHIV taking DTG has been described in sub-Saharan people and can differ in participating countries.

Randomisation will only be performed if study drugs are readily available at the trial site and will take place on the day of inclusion (W0) visit.

The inclusion (W0) visit, which corresponds to ART initiation, will be performed as soon as possible and up to 30 days after the screening visit. It will include a clinical evaluation: weight and BMI, waist and hip circumference and ratio, body temperature, blood pressure, heart assessments (ECG, transthoracic echocardiography, ABPM), liver transient elastography (Fibroscan with CAP module); medical history since the last visit, medication history and concomitant medications, randomisation, ART dispensation, mental health and quality of life questionnaire (EQ-5D-3L, HIVTSQ, DASS-21, PSQI, WHOQOL HIV-BREF); local laboratory tests: CBC, AST and ALT, creatinine blood levels and creatinine clearance calculation, fasting serum lipids (total cholesterol, HDL, LDL, triglycerides), fasting glycaemia, glycated haemoglobin for patients with known diabetes mellitus, urine strip (proteins and glucose), ±urine pregnancy test; frozen samples (whole blood and plasma) for insulin dosage, homeostasis model assessment-estimated insuline resistance (HOMA-IR), virologic analyses, pharmacokinetic and pharmacogenomics; and a metabolic substudy in women, including blood frozen samples (serum) for central analysis (adiponectin, leptin, sCD14, sCD163), adipose tissue biopsy and DEXA scan.

### Safety considerations

In the event of a new diagnosis of hypertension and/or diabetes, the patient will be managed according to national guidelines and all new treatments must be reported. Severe muco-cutaneous events will lead to the immediate decision to discontinue all drugs possibly implicated, including DOR+TDF+3 TC or DTG+TDF+ XTC, and cotrimoxazole. Patients with grade 3 elevation of ALT will be closely followed with weekly assessments, and hepatotoxic drugs or study drugs will be discontinued in case of grade 3 or 4 hepatitis with clinical symptoms. In the case of severe neuropsychiatric disorders, the role of DTG or DOR will be systematically considered, and investigators will also consider discontinuation of these drugs. At the discretion of the investigator, therapy may generally be reinitiated when laboratory abnormalities or clinical adverse events return to near normal or to baseline values. If the adverse event is considered serious or if exposure to the study drug(s) poses additional potential significant risk to the patient, rechallenge is not recommended. A dosing interval adjustment of TDF is recommended in all patients with creatinine clearance of 30–49 mL/min occurring after the screening visit. TDF should be permanently discontinued if creatinine clearance is <30 mL/min; alternatively, a switch to other NRTIs could be considered (zidovudine/3TC, abacavir/3TC). Follow-up after drug discontinuation follows planned trial visits, with an additional visit 4 weeks after drug discontinuation to assess evolution of the event that led to termination of the study drug.

### Sample size and statistical analyses

#### Sample size justification

Our trial aims to demonstrate that DOR-based therapy is non-inferior to DTG-based therapy in terms of virologic success at week 48. We hypothesise that, in these adults initiating ART, 75% of participants who start a DTG-based regimen (and the same proportion with DOR-based regimen) will have plasma HIV-1 RNA levels <50 copies/mL at week 48. Using a non-inferiority margin of −12%, 274 participants are needed in each arm (SAS, proc power, lower confidence limit for difference in proportions, one-sided test, α=2.5%, 1−β=90%). The non-inferiority margin was set at −12%, in line with regulatory guidance for ART trials using virological endpoints (HIV-1 RNA <50 copies/mL), for which margins of −10% to −12% are considered acceptable.^33^ This margin was deemed acceptable as it is a pragmatic academic phrase for a three-trial and not an industry registration trial, and given the expected safety and metabolic benefits of DOR compared with DTG. The margin was defined in the protocol and approved by the trial Scientific Committee and the Data and Safety Monitoring Board/Independent Data Monitoring Committee (IDMC) prior to trial initiation.

To account for participants who do not have the criteria at the end of the trial (lost to follow-up, censored), we will include 305 participants per group using an inflation factor of 1.111.

Regarding metabolic substudy sample size, we plan to enrol 80 women (40 in the DOR group and 40 in the DTG group). This number, based on previous works, is presumed to allow finding differences according to the treatment and other factors and remains achievable during the enrolment period. Inclusions will be monitored by the international Clinical Trial Unit (iCTU) to ensure an equivalent allocation of patients per arm and per site.

#### Population of analysis

All randomised participants should, a priori, be included in the analysis, including participants who died, were lost to follow-up or withdrew from the trial. The Trial Scientific Committee may decide to exclude participants from the analysis. The decision to exclude a participant must be taken while blinded to the participant’s trial arm and his/her outcomes since inclusion. A participant may be excluded from the analysis if s/he meets one of the following criteria: did not initiate the trial treatment to which she/he was randomised (as long as she/he did not know to which group s/he was randomised), withdrew consent or was wrongfully included with respect to major eligibility criteria.

#### Statistical analysis

The primary hypothesis will be assessed based on the proportion of participants achieving HIV-1 RNA levels <50 copies/mL at week 48 using the full analysis set, which should include all randomised participants. As defined by the FDA Snapshot Approach, all missing data will be classified in the category ‘no data’, regardless of the reason: discontinued study due to an adverse event or death or for other reasons, or on study but missing data in window. For the analysis at time points of interest, the difference in proportions between treatment groups and the associated 95% CI will be calculated using the stratum adjusted Mantel-Haenszel method (screening HIV-1 RNA≤100 000 or >100 000 copies/mL and countries). A margin of 12 percentage points is used to define the non-inferiority of DOR 100 mg+tenofovir DF+lamivudine vs DTG-based regimen. DOR 100 mg+tenofovir DF+lamivudine will be concluded non-inferior to DTG-based regimen, if the lower bound of the two-sided 95% CI for the difference in the proportion of participants with HIV-1 RNA <50 copies/mL at week 48 (DOR 100 mg daily minus DTG-based regimen) is greater than −12 percentage points. The non-inferiority margin has been set at −12% as the ANRS0392 EvaLuating the non-inferiority of DORAvirine vs DOlutegravir (ELDORADO) trial is a pragmatic academic phase 3 trial and not an industry registration trial. Moreover, according to the FDA recommendations for industry registration trials, for a virologic endpoint (VL<50 copies/mL), a non-inferiority margin of −12% to −10% remains acceptable.^33^ The aim of the study is to demonstrate non-inferiority in view of the expected benefits of DOR on safety criteria, which justify the choice of a 12% non-inferiority margin.^34^ A sensitivity analysis will be performed where the discontinuation of the study drug due to TB will be considered as a virologic success when the participant achieves virologic success. To determine whether the treatment effect is consistent across various subgroups, the estimate of the between-group treatment effect (with a nominal 95% CI) for the primary endpoint will be calculated and plotted within each category of the following classification variables: (1) age, (2) gender (female, male), (3) countries, (4) baseline HIV-1 RNA categories (≤100 000 copies/mL, >100 000 copies/mL) and (5) baseline CD4 categories.

Provided non-inferiority is established, it can be further concluded that DOR-based regimens are superior to DTG-based regimens if the lower bound of the two-sided 95% CI for the difference in response rate is greater than zero. With our sample size, the study will have at least 85% power to demonstrate that the proportion of patients in virologic success at week 48 is >85% higher in the DOR-based regimen group as compared with the DTG-based regimen group using a two-sided test, α=5%, provided that the proportion of success in the DTG-based regimen group is higher than 75%. In the NAMSAL study,^35^ at week 48, the incidence of obesity was 5.4% in the EFV-based treatment arm and 12.3% in the DTG-based treatment arm. Taking these as hypotheses, we would have a power of 85% to detect such differences between treatment arms with our sample size and a bilateral alpha of 5%.

For the other key safety endpoints, considering that the proportion of key safety events could vary from 1% to 8% in the DOR arm, we would have at least 80% power to detect differences with the DTG arm of at least 7.3%. The treatment differences will be tested between treatment groups for the proportion of participants with the following events: occurrence of obesity at weeks 48 and 96, newly measured HOMA ≥2 at weeks 48 and 96, hypertension newly detected at weeks 48 and 96 and other safety analyses.

No interim analysis is planned unless asked by the IDMC.

Baseline characteristics, curve of inclusions, number of scheduled/applied visits, deviations to the protocol, probability of death, loss to follow-up and morbidity events will be described overall and by randomisation arm. For continuous data, means, SD, medians, IQRs and ranges values will be given. For categorical data, absolute numbers and percentages will be given, and 95% binomial proportion CIs will be calculated; the exact method will be used when appropriate. For time-dependent variables, incidence of the first event of interest per 100 patient-years and Kaplan-Meier probability of occurrence of the first event over time will be estimated with their 95% CIs.

A detailed statistical analysis plan will be written and validated before the database is frozen.

### Handling and storage of data

This trial will be conducted in accordance with ethics principles contained in the World Medical Association Declaration of Helsinki (Ethical Principles for Medical Research Involving Human Subjects) and the ANRS MIE Ethics charter for research in developing countries and with each country’s laws and regulations, as well as with the Good Clinical Practice E6(R2) and Good Clinical Laboratory Practice. The trial is registered at http://www.clinicaltrials.gov/ under the number NCT06203132.

The data will be the subject of computer processing on behalf of the sponsor. The sponsor declared the study database to the French ‘Commission Nationale Informatique et Liberté’ (CNIL, Decision DR-2024-324).

### Patient and public involvement

Community advisory boards (CISPOC Mozambique, TRT5 group in France and the RIP+association in Côte d’Ivoire) reviewed the informed consent form before submission to ethics committees. In Brazil, the Community Advisory Board (CAB) (called CCA: Comitê comunitário Assessor) reviewed the ELDORADO trial protocol before Institutional Review Board (IRB) submissions. In Thailand, the study as well as the consent documents in Thai language have been presented and discussed with the AMS-PHPT CTU CAB (that included members of Mplus Foundation and CAREMAT Foundation) and to the Chiang Rai HIV/AIDS Patient Network.

## Ethics and dissemination

Primary outcome results (week 48) are expected in early 2028. The project was submitted to and approved by national ethics committees and pharmaceutical regulatory authorities in all participating countries and received authorisation from the French National Commission for Data Protection and Liberties (CNIL) under approval number 924 302. Written informed consent is obtained from all participants prior to any study-specific procedures and trial enrolment, in accordance with the Declaration of Helsinki and applicable national regulations. Study findings will be disseminated through publication in peer-reviewed journals and presentations at national and international scientific conferences. Results will also be communicated to policymakers, healthcare professionals, community stakeholders and study participants through appropriate dissemination activities, including policy briefs, stakeholder meetings and lay summaries on dedicated and easily accessible platforms.

## Discussion

The rationale for our randomised trial comparing DOR versus DTG is twofold.

First, this head-to-head trial comparing DOR (or another NNRTI) and the INSTI recommended as first-line ART by the WHO guidelines has not been performed previously. Only indirect comparisons have been conducted, either DOR versus EFV or versus ritonavir-boosted darunavir, or DTG versus EFV. In the DRIVE-AHEAD phase III, double-blind trial, comparing DOR to EFV in ART-naive PLHIV, non-inferiority was demonstrated at week 48 with significantly lower rates of neurological adverse events and a better lipid profile^24^; in DRIVE-FORWARD, comparing DOR to ritonavir-boosted darunavir in ART-naive PLHIV,^11^ non-inferiority was demonstrated at weeks 48 and 96. Three RCTs have demonstrated the non-inferiority of DTG compared with EFV. In the SINGLE double-blind trial in treatment-naïve PLHIV, the superiority of the DTG arm was primarily driven by fewer discontinuations due to adverse events.^36^ At week 96, the superiority of DTG was confirmed.^37^ In the open-label phase III ANRS 12313 NAMSAL trial, the non-inferiority of DTG was demonstrated at weeks 48 and 96. Week 192 outcomes confirmed a higher proportion of viral suppression in the DTG arm.^14^ In the phase III, investigator-led, open-label, ADVANCE trial, non-inferiority was established at weeks 48 and 96.^7 8^

Second, recent cohorts and trial data in PLHIV following ART initiation suggest that DTG is associated with higher weight gain,^5^ higher incidence of obesity,^6^ onset of long-term type 2 diabetes mellitus and hypertension^16 30 38^ than other regimens. These non-communicable diseases already have a major public health impact in both developed and developing countries. Over the past three decades, both life expectancy and healthy life expectancy in the USA have declined in global rankings,^39^ with obesity and overweight contributing to substantial morbidity and mortality: 11.6 million disability-adjusted life-years and 335 000 deaths in 2021 in the USA were attributed to overweight and obesity.^40^ In 2022, an estimated 828 million (95% CI 757 to 908) adults had diabetes, an increase of 630 million (554 to 713) from 1990. From 1990 to 2022, the age-standardised prevalence of diabetes increased in 131 countries for female subjects and in 155 countries for male subjects. The largest increases were in low-income and middle-income countries in south-east Asia, south Asia, the Middle East and north Africa, and Latin America and the Caribbean. In 2022, age-standardised treatment coverage was the lowest in countries in sub-Saharan Africa and south Asia, and treatment coverage was less than 10% in some African countries.^41^ Initially, studies involving persons with obesity and pre-diabetes investigated whether lifestyle interventions, pharmacotherapeutics or bariatric surgery prevent or delay the onset of type 2 diabetes. Overweight and obesity are independently associated with an increased risk of cardiovascular events, even after the influence of metabolic cardiovascular risk factors linked to excess weight has been accounted for^42^,^45^ and with a rise in heart failure prevalence. Weight-loss interventions ameliorate systemic inflammation, decrease epicardial adipose volume, reduce the risk of incident heart failure and alleviate symptoms in patients with established heart failure with preserved ejection fraction.^46^,^49^ In particular, the class of GLP-1 receptor agonists, mainly semaglutide, has shown efficacy not only in the treatment of type 2 diabetes, but also in obesity and heart failure.^50^,^52^ However, while these results are promising, their real-world application may be limited in the context of the ELDORADO trial, which includes countries in Latin America and sub-Saharan Africa, where access to GLP-1 receptor agonists remains extremely limited due to high costs and availability issues. Therefore, although relevant from a mechanistic and clinical perspective, the feasibility of using such pharmacologic interventions in these settings is currently low. Obesity is a risk factor for MASLD (formerly known as non-alcoholic fatty liver disease), which can progress to MASH (formerly non-alcoholic steatohepatitis), a coexisting condition in many individuals with MASLD.^53^ The prevalence of MASH is increasing globally in parallel with obesity and type 2 diabetes mellitus.^54 55^ Steatotic liver disease affects around 30% of the global population and is mainly driven by obesity, type 2 diabetes and alcohol intake.^56^ MASH is associated with an increased risk of cardiovascular disease and can convey a higher risk of liver-related complications and death. The beneficial effects of weight reduction on MASLD and MASH are well documented.^54 57 58^ Obesity is also a major risk factor for the development and progression of osteoarthritis of the knee^59^,^61^ and sleep apnoea.^62^,^64^

Unlike NAMSAL, ADVANCE and OPTIDOR—which are/were conducted exclusively in sub-Saharan Africa and included only African participants—this trial will enrol a geographically and ethnically diverse population across four continents (Latin America, Europe, sub-Saharan Africa and Asia). This broader recruitment will allow a more comprehensive evaluation of virological efficacy and metabolic outcomes, such as weight gain and obesity, across different ethnic and regional backgrounds. Moreover, by including pharmacokinetic and pharmacogenetic substudies in three sub-Saharan African countries, this trial will address critical knowledge gaps not explored in the previous studies.^65^ In addition, ELDORADO will provide a critical contribution to documenting other potential toxicities, including organ-specific adverse events, mental health outcomes and quality of life, by generating robust data from a geographically and ethnically diverse population within a rigorously conducted clinical trial.

Recruiting both male and female participants, with TDF in both arms (as TAF has also been associated with weight gain, though perhaps via different mechanisms than INSTIs),^19^ and including an ancillary metabolic substudy in women, will allow a precise definition of the impact of DTG. This design will also help explore the pathophysiology of weight gain and obesity. The other ongoing head-to-head comparison of DOR versus DTG in treatment-naïve PLHIV-1 conducted in South Africa, the OPTIDOR trial, has a different NRTI backbone in each arm: TAF/FTC with DTG and TDF/FTC with DOR. The results of that study will be complementary to our trial. However, as only TDF/XTC is currently recommended by WHO as the NRTI backbone, our results might be more relevant for low-income and middle-income countries.

By including a diverse, multicontinental population and focusing on key long-term outcomes such as overweight and obesity, this trial is uniquely positioned to inform clinical practice in both high-resource and low-resource settings. Its findings may provide the necessary evidence to support the inclusion of DOR as a first-line alternative in future WHO guidelines for HIV treatment.

This study has several strengths and limitations that should be considered when interpreting its findings. A major strength is the inclusion of participants from ethnically diverse populations across Latin America, sub-Saharan Africa, Europe and Asia, a diversity that remains underrepresented in conventional phase III randomised trials but is essential to improve the generalisability of both effectiveness and safety outcomes. In addition, the use of TDF rather than tenofovir alafenamide fumarate in both treatment arms is expected to facilitate a clearer assessment of the specific contribution of the third agent (DOR or DTG) to weight gain, by limiting confounding related to background therapy. However, some limitations should be acknowledged. Furthermore, selecting a therapeutic regimen based on TDF instead of TAF allows evaluation of the WHO-recommended regimen among ART-naïve PLHIV.

Although follow-up extends to 2 years, this duration may be insufficient to fully characterise long-term virological durability and metabolic outcomes, including sustained weight changes and cardiometabolic complications, which will require longer-term observation.

## Supplementary material

10.1136/bmjopen-2025-110560 online supplemental file 1
