# Supplementary material for: Doravirine versus dolutegravir-based regimen in antiretroviral treatment-naive people living with HIV-1 (ANRS0392s ELDORADO): protocol for an international, open-label, randomised, non-inferiority, phase III trial
Source: BMJ Open. 2026 Feb 5;16(2):e110560. doi: 10.1136/bmjopen-2025-110560 (PMC12878352; doi:10.1136/bmjopen-2025-110560)
Supplement: online supplemental file 1 [file bmjopen-16-2-s001.pdf]

Information Letter

ANRS0392s ELDORADO

Phase III, open-label, randomized, multicenter trial **E**va**L**uating the non-inferiority of **DORA**virine versus **DO**lutegravir based antiretroviral regimens in treatment-naïve people living with HIV-1 infection

EU-CT number: 2023-508626-10-00
Clinicalstudys.gov identifier: NCT06203132
Version n°2.0 of 21/05/2025 approved by ethics committee <name of EC> on xx/xx/xxxx

The study's coordinating investigators are:

- **Pr Beatriz GRINSZTEJN (INI/Fiocruz, Rio de Janeiro, Brazil)**
- **Dr Pierre SELLIER (Infectiology Department, GH Saint-Louis-Lariboisière-Fernand Widal, Paris, France)**

The study sponsor is Inserm-ANRS MIE, represented by the Director of ANRS Maladies infectieuses émergentes (Emerging Infectious Diseases), Pr Yazdan YAZDANPANAH.

ANRS MIE is located at PariSanté Campus, 2 rue d'Oradour-sur-Glane, 75015 Paris, France.

- **This note is intended to help you decide whether or not to participate in the study described below.**
- **You have the right to take time to think about and discuss this study, and to ask the study doctor any questions you may have.**
- **If you wish, you can call on an impartial witness to help you decide whether or not to take part in this study, and throughout the study process if necessary.**
- **You are free to answer "yes" or "no" to the question: "Do you wish to participate in this study?"**
- **If you agree, you can change your mind at any time and ask to no longer take part in this study. We only ask that you inform the study doctor of your decision as soon as possible.**
- **If you no longer wish to participate, you will continue to receive the best possible care.**
- **You may be asked to sign a new consent form if the study is modified and/or extended.**

Words underlined in the text are explained in the glossary.

GLOSSARY

Antiretroviral therapy/treatment (ART): treatment to block certain stages in the development cycle of one or more viruses.

Collection of biological samples: set of samples taken from people taking part in a study (e.g. blood, cells, etc.) and prepared for analysis and preservation.

Consent form: document by which you declare that you are aware of the terms of participation in a research project and give your agreement to take part in it.

Coordinating investigator: doctor or qualified person who supervises the conduct of the study at the various participating sites.

Data (personal): any information about you collected as part of the study.

HIV viral load: number of HIV copies circulating in the blood.

Impartial witness: person independent of the study designated by the participant, who may be a parent, a relative or the attending physician, and who will be consulted in the event that the participant is unable to express his or her wishes and receive the necessary information to do so. If you are unable to express your consent in writing, it may be attested by your impartial witness. If you wish, your impartial witness can accompany you through the process and attend medical appointments to help you make decisions during the study.

Inclusion: when all the conditions are right for you to take part in the study.

Non-inferior efficacy: a non-inferiority trial seeks to demonstrate that the efficacy of a new treatment is at least as good as that of a reference treatment.

Pharmacokinetics: study of drug dosage in blood or other tissues. These studies help to determine whether drug doses are appropriate.

PLHIV: people living with HIV

Randomization: a method of randomly assigning one of the study treatments to a group of people, allowing comparisons of treatments/strategies/approaches.

Sponsor: legally and financially responsible party for the study. The sponsor is also responsible for processing your data.

Study doctor: the qualified person who will be following you as part of the study. This may be your regular physician, another physician involved in the study or another qualified person.

Treatment-naïve person: person who has never received any treatment for a disease.

TABLE OF CONTENTS

1. STUDY CONTEXT AND OBJECTIVES 3
2. STUDY PARTICIPATION CONDITIONS 3
3. STUDY DESIGN 4
4. YOUR SAMPLES 6
5. STUDY TREATMENTS 7
6. SEXUALITY, CONTRACEPTION AND PREGNANCY 8
7. CONSTRAINTS RELATED TO PARTICIPATION IN THE STUDY 9
8. FORESEEABLE RISKS AND BENEFITS 9
9. REACCESS TO INFORMATION AND RESULTS 10
10. DATA PROCESSING AND RIGHTS ASSOCIATED WITH YOUR PARTICIPATION 10
11. STUDY APPROVAL 13
12. INSURANCE 13
13. CONTACT 13

A consent form is on the last page: this signed document attests to your willingness to take part in the study, and contains the contact details of the study doctor.

Dear Sir/Madam,

The study doctor invites you to participate in the ANRS0392s ELDORADO study *"Phase III, open-label, randomized, multicenter trial **E**va**L**uating the non-inferiority of **DORA**virine versus **DO**lutegravir based antiretroviral regimens in treatment-naïve people living with HIV-1 infection*".

1. STUDY CONTEXT AND OBJECTIVES

To date, in 2022, the World Health Organization (WHO) estimated that 39 million people were living worldwide with Human Immunodeficiency Viruses (HIV).

Antiretroviral therapy (ART) blocks the multiplication of HIV in the blood (assessed by the HIV viral load) and therefore improves immune defenses (assessed by the number of CD4 cells). On treatment, people living with HIV are less susceptible to complications of HIV.

Since 2018, the reference ART recommended by the WHO for people living with HIV-1 who have never been treated before (also called "treatment-naïve") has been dolutegravir. Although this treatment has proven its effectiveness, recent studies have reported problems with how the body uses food and energy ("metabolic disorders") associated with its use, notably weight gain, high blood pressure and diabetes.

Doravirine is a new drug that appears to be very well tolerated. It is also recommended as an alternative treatment in the recommendations of the International Antiviral Society-USA (IAS-USA), and is indicated as a first-line treatment by the recommendations of the European AIDS Clinical Society (EACS). In various parts of the world, it is therefore one of the first-line treatments offered to patients diagnosed with HIV-1.

Although doravirine efficacy has already been compared with several antiretroviral drugs, it has not yet been compared with dolutegravir in treatment naive people.

The aim of this study is to show that doravirine-based ART is non-inferior to dolutegravir-based ART. The benefit of this combination would be to reduce dolutegravir-related side effects, notably moderate weight gain shown in patients receiving dolutegravir. If the results of this study are conclusive, the doravirine-based therapy could be extended and recommended for treatment-naive people with HIV-1 infection.

The primary objective is to determine whether participants on doravirine have a non-inferior response to their treatment than those on dolutegravir. This non-inferiority will be determined after 48 weeks (11 months) of treatment by comparing HIV-1 viral load in the blood according to the treatment taken.

Secondary objectives of the study include comparison of HIV-1 levels in the blood between both treatments after 96 weeks of treatment (22 months), assessment of treatment resistance in the event of treatment failure, and side effects (notably weight gain, hypertension or diabetes).

2. STUDY PARTICIPATION CONDITIONS

If you decide to take part in this study, an initial visit (called a "pre-inclusion visit") is scheduled up to 2 to 4 weeks (up to 4 weeks in France, up to 2 weeks in Brazil, Cameroon, Côte d'Ivoire, Mozambique or Thailand) before the start of the study treatment. This is to check that you are eligible to take part in the study. The study doctor will assess your health status (consultation, clinical examination, weight and height measurements, blood tests, chest X-ray) to determine whether you meet the criteria for participation in the study.

The main criteria for participation, are as follows:

- Being 18 years of age or older
- Having never received antiretroviral treatment
- Having been diagnosed with HIV-1
- Having signed the consent form at the end of this document. This form must be signed before any examination (e.g. blood test, X-ray, etc.) is carried out as part of the study
- Not taking contraindicated medications (certain anti-infectives, psychotropic drugs or anti-epileptics), which will be checked by your doctor
- Not having active tuberculosis

- Not being pregnant or not breastfeeding
- If you are a person capable of becoming pregnant, you will be asked to use contraception throughout the study; if your partner(s) is/are capable of becoming pregnant, you will be asked to protect yourself during intercourse as to protect your partner.
- Not being under judicial or administrative protection
- Not participating in another study during the study period.

Your study doctor will check all the criteria applicable to this study to validate your participation. If you meet the criteria for participation, you can be definitively included in the study. If it turns out that certain conditions do not allow you to participate in the study, your study doctor will explain the reasons and decide with you the best medical care.

If you agree to be included in the study, you will be asked to sign an agreement form that will include information about the study: an “informed consent form”. At any time during the study, you will have the right to withdraw your consent without justification. Your study doctor will then decide with you how best to proceed.

3. STUDY DESIGN

The study will enroll 610 participants in several hospitals in 6 countries (Brazil, Cameroon, Côte d'Ivoire, France, Mozambique and Thailand).

The duration of your participation in the study is 96 weeks (around 22 months, a little less than 2 years).

The total duration of the whole study is at least 3 years. This includes the time needed to include all participants, and the time needed for each participant to be followed up until the last participant's last visit.

At the end of your participation, you will be able to discuss your results and your experience during the study with your study doctor (see section “9. Reaccess to information and results”). The results of the study will be made available.

Participants are randomly divided into 2 groups. You have as much chance of being assigned to one group as to the other:

- **Group 1: Doravirine arm**: Participants receive doravirine (100mg), tenofovir disoproxil fumarate (300mg) and lamivudine (300mg) as a single tablet for 96 weeks,

Or

- **Group 2: Dolutegravir arm**: Participants receive dolutegravir (50mg), tenofovir disoproxil fumarate (300mg) and emtricitabine (200mg) or lamivudine (300mg) as <XX> tablets for 96 weeks**.**

Randomization is the best way to compare the efficacy of treatment between the two groups. You should know that neither you nor your doctor will be able to choose the antiretroviral treatment you’ll receive.

Visits calendar
The schedule below gives details of the consultations, examinations and samples you will be given at each visit.

For the success of the study and for your own safety, it is important that you follow the schedule of visits as closely as possible. If you are unable to attend a study visit, please let your doctor know as soon as possible so that you can work together to find a solution.

Visits / Examinations	Pre-inclusion W-4/W-2*	W0	W4	W12	W24	W36	W48	W72	W96	Virological failure visit
**Signature of informed consent**	X									
Consultation and clinical examination[a]	X	X	X	X	X	X	X	X	X	X
Chest X-ray	X									
Liver assessment (Fibroscan)		X					X		X	
Heart assessments (ECG, echocardiography, 24-hours blood pressure monitoring)		X					X		X	
Questionnaires		X			X		X		X	
Bring back ARV drugs/bottles at study visit			X	X	X	X	X	X	X	X
Come before daily drug intake			X		X					
**Samples for all patients**										
Complete Blood Count[b] / Biochemistry[c]	X	X	X	X	X		X		X	
CD4/CD8	X	(X)[f]					X		X	
Lipid profile and fasting blood glucose[d]		X			X		X		X	
Glycated hemoglobin[e]		X			X		X		X	
Discriminant HIV test documented	X									
Genotypic resistance testing	(X)[f]	X[g]								X[g]
HIV-1 RNA viral load	X	(X)[f]	X	X	X	X	X	X	X	X
Urine pregnancy test[h]	X	X	X	X	X	X	X	X	X	
Urine dipstick		X					X		X	
**Biological sample collection**										
Dried Blood Spots			X[i]	X	X[i]	X	X[i]	X	X[i]	X
Plasma		X	X[i]	X	X[i]	X	X[i]	X	X[i]	X
Whole Blood		X								
**Maximum blood volume**	30 mL	60 mL	20 mL	20 mL	35 mL	10 mL	50 mL	10 mL	50 mL	20 mL
**Maximum number of tubes**	6	12	4	4	7	2	10	2	10	4

*: W-4 in France, W-2 in Brazil, Cameroon, Côte d'Ivoire, Mozambique and Thailand
a: Measure of height (only at 1st visit), weight, blood pressure, waist and hip circumference (only at W0, W24, W48 and W96).
b: Hemoglobin, white blood cells, red blood cells, platelets, granulocytes, monocytes, lymphocytes
c: Transaminases (ALT, AST), creatinine, creatinine clearance
d: After a fasting period of at least 12h
e: Only for participants with diabetes mellitus
f: Only if recommended by local health authorities
g: Genotypic resistance test to be performed at screening locally and in case of Virological Failure on VF and baseline frozen samples
h: For people of childbearing potential only
i: Sample before daily drug intake

W-4/W-2 corresponds to the pre-inclusion phase, which lasts a maximum of 2 to 4 weeks (2 weeks in Brazil, Cameroon, Côte d'Ivoire, Mozambique and Thailand, and 4 weeks in France) and during which the examinations necessary to determine your eligibility are carried out.

W0 (Week 0) corresponds to the 1st day of treatment. During the visit, a routine check-up is carried out (measure of the amount of virus and of CD4/CD8 cells in blood, complete blood count, liver and kidney parameters check, lipid levels measure). A chest X-ray is done to ensure that you do not have active tuberculosis. Liver and heart assessments are carried out to measure your baseline status, and verify your potential liver and heart diseases risks. Blood samples (whole blood and plasma) will be taken and frozen to build up a collection of biological samples for further analysis. The total amount of blood taken will be a maximum of 60 mL.

Monitoring

During your follow up, if your HIV viral load shows that the treatment is not effective enough, you will be asked to come back as soon as possible and within 4 weeks to perform a “virological failure visit” and repeat the tests. If treatment failure is confirmed, meaning that the treatment was insufficiently effective, a genotypic resistance testing will be performed. You will continue to be monitored as part of your routine care and your study doctor will decide what treatment may best treat your infection.

During your follow-up, if your state of health deteriorates or if you experience any undesirable side effects to your treatment, it is important to inform your study doctor and discuss with them as soon as possible.

Throughout the study process, scientific oversight committees monitor the efficacy of treatments and the occurrence of adverse events. These committees ensure that the study proceeds smoothly, and in particular that the safety of participants is guaranteed. If necessary, for safety reasons for example, they may decide to modify or stop the study.

You will be informed if any change occurs during your follow-up and may be asked to sign a new consent form.

Possible Medical Alternatives

The treatments in this study program already have marketing authorization in various countries and are among the 1st-line treatments for patients diagnosed with HIV-1 who are naive to antiretroviral therapy.

This means that you are under no obligation to take part in the study in order to benefit from active treatment for HIV. Your decision to participate in this study must be free and voluntary.

4. YOUR SAMPLES

1) Use during study

As part of the study, you will have routine blood samples taken at visits W-4/-2, W0, W4, W12, W24, W36, W48, W72 and W96. A maximum of twelve (12) tubes (60mL) will be drawn for the main study at W0. These samples will be used to carry out blood tests and to build up a collection of biological samples for analysis as part of this study (fasting insulin dosage, HOMA index to calculate insulin resistance, or pharmacokinetic analysis). These analyses will focus in particular on your biomarkers to obtain a better understanding of your disease and the effects of the treatment. Specific consent for further use of your samples is requested on the consent form.

If necessary, your samples can be stored for later use (see next paragraph). Each sample is labelled and numbered in a coded way (neither your last name nor your first name will appear) and they are said to be "pseudonymized".

Sampling schedule

The sampling schedule is summarized in the table present in section “3. Study Design”.

During the course of the study, your samples will be transferred to the ANRS MIE biobank located in Centre de Recherche Biologique (Biological Research Center) in Bordeaux, France, for centralization, then dispatched to the various laboratories involved in the study according to the analyses planned.

2) What happens to your samples after the study is completed?

Throughout the course of the study, samples will be taken from you and might be used for further research if you agree to it in the informed consent form.
If any of your samples are not used, they will be, at your request:

- Either destroyed
- Or stored at the Centre de Ressources Biologiques (Biological Research Center) in Bordeaux University Hospital, France, for future scientific research on HIV and its associated comorbidities, and emerging infectious diseases.

They may then be reused, or even transferred, for the benefit of public or private research teams in France or abroad. As this collection of biological samples will be stored and declared in France, French law applies and thus, you will not receive any remuneration for this reuse or handover. As during the study, your samples and data will remain coded, and neither your name nor your first name will be divulged.

The consent form given to you will allow you to express your wishes. Your consent does not prevent you from subsequently:

- Withdrawing your consent to any further use,
- Objecting to a particular re-use.

3) Genetic study

As genetic study is planned during this study, you will be informed of these analyses through a separate information letter. If you consent to it, you will have to indicate it in the specific consent form.

4) Your rights

You have the same rights to your samples as those mentioned in section "10. Data processing and rights associated with your participation". Your decision will not affect your participation in this study or your medical care.

The results of the research carried out on your remaining samples may be linked to your medical data collected as part of ELDORADO study.

5. STUDY TREATMENTS

uring randomization, you will be assigned to one of two study groups:

Countries with single tablet regimen for dolutegravir:

	Molecule name	Form	Dosage	Recommendations
**Doravirine group**	**Doravirine**<br>Tenofovir disoproxil fumarate<br>Lamivudine	Tablet	1 oral tablet per day	Take with or without food
**Dolutegravir group**	**Dolutegravir**<br>Tenofovir disoproxil fumarate<br>Lamivudine	Tablet	1 oral tablet per day	Take with or without food

Other countries:

	Molecule name	Form	Dosage	Recommendations
**Doravirine group**	**Doravirine**<br>Tenofovir disoproxil fumarate	Tablet	1 oral tablet per day	Take with or without food

	Lamivudine			
**Dolutegravir group**	**Dolutegravir**	Tablet	1 oral tablet per day	Take with or without food
	Tenofovir disoproxil fumarate<br>Emtricitabine		1 oral tablet per day	Best taken with food

Group 1 (Doravirine arm):

MK-1439A: what you need to know

It is marketed by the MSD laboratory.
Doravirine is a potent and relatively novel non-nucleoside reverse transcriptase inhibitor (NNRTI) for treatment of HIV-1 infection in people living with HIV having never received ART.
Its use has been documented and it is a safe and well tolerated treatment as doravirine is recommended as first line ART by European AIDS Clinical Society Guidelines and as an alternative to integrase strand transfer inhibitor (INSTI) in International Antiviral Society–USA Guidelines.
The main side effects you can expect are nausea and headaches.
If you wish to know more, as the medicine is already in use in North America and Europe, the Summary of Products Characteristics are available at:
- For North America: https://www.accessdata.fda.gov/drugsatfda_docs/label/2018/210807s000lbl.pdf
- For Europe: https://ec.europa.eu/health/documents/community-register/2018/20181122142677/anx_142677_en.pdf

Group 2 (Dolutegravir arm):

What you need to know

Dolutegravir is an integrase strand transfer inhibitor (INSTI) which has been extensively studied and its safety and efficacy have been demonstrated. It is one of the reference ART. Its use has been recommended by the most recent World Health Organization (WHO) international guidelines for all population of people living with HIV as 1st and 2nd-line treatment.
The main side effects you can expect are nausea, diarrhea or headaches.
If you wish to know more, you can refer to the Summary of Products Characteristics that will be provided with the medicine or ask for it to your study doctor.

We ask you to return empty blister packs, any remaining medication and any boxes of your own medication, whether empty or not, at each visit.

At W4, W24, W48 and W96 you should come before taking your treatment this day.

Taking other medications during the study: TALK TO YOUR STUDY DOCTOR FIRST.

It is important to inform your study doctor of any medications (prescription or non-prescription) you are taking or intend to take during the study. Some treatments are not allowed during the study because they cannot be taken together with ART. The use of certain medications such as some antivirals (e.g., aciclovir, valaciclovir…) is not recommended during the study and as such should be taken with caution. You should therefore inform your study doctor of all medications you are taking to check whether they are compatible with the treatments of the study.

The following drugs are strictly not allowed under any circumstances during the study:

- anti-infectives: *rifabutin, rifampicin/rifampin*;
- psychotropics/anti-epileptics: *phenytoin, phenobarbital*;
- high-dose non-steroidal anti-inflammatories: *diclofenac, ketoprofen…*;
- antibiotics: *gentamicin...*;
- immunosuppressants: *tacrolimus, sirolimus.*

If you absolutely need any of these treatments for your health, your study doctor must be immediately informed for appropriate decisions with regard to your study treatment.

Any dietary supplements, herbal teas or other phytotherapy products **containing St. John's wort (*Hypericum perforatum*) or grapefruit are also forbidden**: as the effectiveness of the study drugs would be altered.

6. SEXUALITY, CONTRACEPTION AND PREGNANCY

Taking your ART daily as scheduled is very important, as people with HIV achieving and keeping an undetectable level of HIV in the blood cannot sexually transmit the virus to their partners. However, HIV antiretroviral treatment does not prevent the transmission of sexually transmitted infections. Moreover, the risk of infecting a partner with HIV is even higher when antiretroviral treatment is stopped. That's why it's so important to use a condom during sexual relations.

As the study drugs are already marketed in various countries, there are Summaries of Product Characteristics (SmPC) for each of these products that contains available information on the effects of the study treatments on pregnancies and newborns. However, you should not plan to become pregnant while taking part in this research.

There is insufficient information on the risks of embryonic and fetal development malformations in people taking doravirin-based treatment during pregnancy, and on the effects on newborn babies.

Data analyzed from the Antiretroviral Pregnancy Registry do not indicate an increased risk of major birth defects in over 600 women exposed to dolutegravir during pregnancy. Thus, this drug is not contraindicated during pregnancy even if data are currently insufficient to address the risk of neural tube defects.

It is not known whether these drugs in semen can cause malformations in the human embryo. You and your partners should be careful to use effective contraception throughout the course of treatment and for up to 7 months after discontinuation.

If, despite all your precautions, you discover that you or your partner is pregnant during the study, you should inform your study doctor immediately.

If you receive doravirine while pregnant, you will be switched to another drug alternative that is deemed safer to be taken during pregnancy in line with national recommendations.

If despite your best efforts, your partner becomes pregnant during the study, we will provide a consent form to ask to collect the details of the pregnancy and of the child to-be-born.

7. CONSTRAINTS RELATED TO PARTICIPATION IN THE STUDY

If you agree to take part in this study, it means that you accept the constraints of the consultations and examinations presented in the schedule. Do not hesitate to ask your study doctor any questions you may have. At each consultation, s/he can give you information concerning your follow-up in the study.

During this study, you will have more blood samples taken than during a usual treatment.

A complete liver and heart check-up will be done, using non-invasive procedures. A Fibroscan (liver echography) will be done to assess the state of your liver; an electrocardiogram (ECG), an echocardiogram (heart echography) and a 24-hours blood pressure monitoring (ABPM) using a Holter will be done to assess the state of your heart. These measures will be repeated each year to monitor the evolution of the measures throughout the research. The constraints of these procedures involve:

- A 3-hours fasting period before your Fibroscan examination.

- Wearing a Holter monitoring tool for 24 hours to monitor your blood pressure throughout the day and the night. It will not be visible but it will inflate regularly and might hinder your sleep. You will have to be careful not to wet it, and you will be asked to come back the next day so it can be removed.

You will also be asked to fill a few questionnaires at some visits regarding your quality of life and your mental health. The information you provide in these questionnaires is for research purposes only and will remain strictly confidential. Some of the questions are personal; you can choose not to answer these if you wish.

8. FORESEEABLE RISKS AND BENEFITS

Risks

The main adverse effects you can expect when participating in this study are nausea and headaches if you are part of the doravirine arm, or nausea, diarrhea or headaches if you are part of the dolutegravir arm.

The treatments used in the study must help to control the virus (i.e. maintain the amount of virus in the blood at a very low level - undetectable viral load). However, there is a risk of treatment failure (persistence of a high quantity of HIV virus in the blood).

To minimize the risk failure may have on your health, the viral load (quantity of HIV in the blood) will be monitored very regularly. In case of treatment failure, a test of susceptibility of the HIV virus to antiretrovirals will be performed and provided to your study doctor, so that you may discuss with them possible alternatives.

As described above, blood samples will be taken. The risks of blood sampling are pain, bruising and, rarely, infection. Blood samples will be taken by experienced staff members.

A chest X-ray will also be taken during the pre-inclusion period to rule out active tuberculosis. The risks of radiography include exposure to a very small amount of radiation to create the image. The amount of radiation from a chest X-ray is very small and comparable to that from many sources of radiation in our everyday environment.

The procedures involved in your liver (Fibroscan) and heart (ECG, echocardiogram, ABPM) check-up do not involve any foreseeable risks. They are all non-invasive procedures that do not require any injection.

Benefits

For the duration of your participation in the study, your medical follow-up will be more frequent than if you were not taking part in the study, allowing you to benefit from a more regular assessment of your state of health.

Direct financial compensation, transportation and meal costs might be covered depending on national practices.

Your participation gives you access to an innovative 1-tablet-a-day treatment, while becoming a player in a study project whose results will benefit others by improving knowledge of HIV and its treatment.

Finally, you will have a right to information on the results of the study.

9. REACCESS TO INFORMATION AND RESULTS

You have the right to be informed, during or at the end of the study, of any information concerning your health held by your study doctor or, where applicable, the doctor or qualified person representing him or her. At the end of the study, you can discuss your results and your experience during the study with your study doctor.

The overall results of the study (results for all participants) will be communicated to participants after the end of the study, via the study doctor.

They will also be available on the Inserm-ANRS MIE website (currently **www.anrs.fr**) in a section dedicated to participants and clinical studies.

At the end of the 96-week study period, regardless of which group you were in, you will receive dolutegravir-based treatment as standard of care. At the end of the study or in the event of premature termination, you will continue to be cared for by the doctor of your choice, and treated in the best possible way in the light of current knowledge, the results of your resistance tests and your treatment history.

10. DATA PROCESSING AND RIGHTS ASSOCIATED WITH YOUR PARTICIPATION

The sponsor of the study, Inserm-ANRS MIE, is responsible for processing your data. The processing of your data, which is necessary for carrying out the study, corresponds to the performance of a mission of public interest entrusted to Inserm (Institut National de la Santé et de la Recherche Médicale – National Institute of Health and Medical Research) (legal basis for processing).

1) Nature of the relevant categories of personal data

The processing of your data during ELDORADO study will allow the results of the study to be analyzed in the light of its objectives (purposes of processing). Your directly identifying data (in particular your first and last names) will be accessible to the study doctor, representatives of the sponsor (Inserm-ANRS MIE), government regulatory agencies, auditors and inspectors in order to ensure that the study has been conducted correctly. All these people are bound by professional secrecy and will be required to implement the necessary measures to preserve the confidentiality of your file. For all other recipients, your data are coded: neither your surname nor your first name will appear and they are transmitted confidentially using a code.

Medical data concerning you, data relating to your life habits (such as drug, alcohol and tobacco use), your ethnic origins, your sexual orientation and information about your quality of life will be collected insofar as they are necessary for the study.

Most of the data required to carry out the study will be collected from your medical records and from the paper documents you fill in (e.g. treatment records or questionnaires). This data will be accessible to the persons mandated by Inserm-ANRS MIE, the sponsor, to ensure the quality of the study.

2) Recipients of your personal data

Research data and statistical analyses are undertaken by the study team (Global Heath in the Global South - GHiGS team, University of Bordeaux, France). Your data is stored by a data hosting server located in University of Bordeaux.

Biological analyses are carried out by laboratories specialized in their respective fields (immunology, virology, etc.).

Inserm-ANRS MIE has a regulatory obligation to transmit safety data (descriptions of adverse events that may occur, including those concerning you) to MSD laboratory that develops and provides doravirine, one of the experimental product used in the study. This ensures the safety of people exposed to the product during clinical study, anywhere in the world.

As part of this study, your samples and data will be transferred to various research laboratories based in Paris, France (cf section "4.1 Your samples – Use during study").

The processing of the study was provided after the establishment of a contract between Inserm-ANRS MIE and the MSD laboratory. As such, your data collected in the context of the study, once pseudonymized, may be potentially used by MSD France or other companies in the group, which commit to take all the technical, legal and administrative precautions necessary to guarantee their protection.

In addition, your personal data, once pseudonymized, may be used by MSD France or directly by its parent company Merck & Co. Inc (Rahway, New Jersey, United States of America) or its affiliates, who will take all necessary technical, legal and administrative precautions to ensure its protection.

3) Transfer of personal data

For research purposes, your pseudonymized data may be transferred to recipients outside the European Union.

Indeed, MSD France, the supplier of doravirine for the study will collect safety data throughout the study. Its parent company Merck & Co. Inc is based in Rahway, New Jersey, USA, and the USA do not benefit from a level of protection comparable to that of the countries of the European Union (GDPR).

However, the protection of your personal data will be guaranteed by a contract between Inserm-ANRS MIE and the recipient, including the standard clauses adopted by the European Commission. If you wish, a copy of the contract/agreement will be made available to you via your study doctor.

When results of the study are published, some of your pseudonymized data will be made available to the scientific community (i.e. journals or researchers). This data will be shared on a secured platform named Recherche Data Gouv, hosted in France (https://recherche.data.gouv.fr/fr).

These data will be made available through controlled access to scientific journals and other researchers and access will have to be formally requested to the sponsor by applicants and data will be shared after consultations with coordinating investigators. The re-use of data will be contractually defined between the sponsor and the re-user.

All protective safeguards will be applied in compliance with the French Data Protection Authority (CNIL) prior sharing.

4) Re-use of your data for further research

Your data may be further processed in connection with HIV and its associated comorbidities, as well as emerging infectious diseases. You have the option of accepting or refusing the principle of this re-use in the consent form provided to you.

If you agree, your data can be reused for further research.

For more information on these new study projects, visit the ANRS Maladies infectieuses émergentes (Emerging Infectious Diseases) website (currently **www.anrs.fr**) in a section dedicated to participants and clinical studies.

Your consent does not prevent you from subsequently:

- Withdrawing your consent to any further use,
- Objecting to a particular re-use.

5) Your rights and how to exercise them

In accordance with the provisions of French Law No. 78-17 of January 6, 1978 as amended relating to data processing, files and freedoms and the General Data Protection Regulation (GDPR - Regulation (EU) 2016/679), you have:

- The right to access your personal information in order to verify its accuracy and, if necessary, to rectify, complete or update it,
- The right to object at any time to the transmission of your data and to have your data removed from future collection. If you exercise this right, your participation in the study will cease,
- The right to restrict their use (right to restriction): the right to temporarily block the use of your data: no operations can be carried out on it.

Due to the nature and legal basis of the processing, you do not have the right to portability of your data, which corresponds to the possibility of recovering your data in a readable and suitable format.

You may withdraw your consent to the study at any time without giving any reason. No new data will then be collected.

Please note, however, that data collected prior to your objection or withdrawal of consent may continue to be treated as confidential, unless you object to it as deleting them would make it impossible or would compromise the achievement of the study objectives.

This doesn't apply to data collected by pharmacovigilance (adverse events) as their processing is based on compliance with a legal obligation: you do not have the "right to object" to collection, the "right to erasure" of said data, nor the right to data portability, which is the possibility of recovering your data in a readable and suitable format.

These rights may be exercised with the study doctor who is monitoring your study and who alone knows your identity. You may also access all your medical data directly, or through the intermediary of a doctor of your choice (attending physician), in accordance with the provisions of article L.1111-7 of the French Public Health Code.

If you have any difficulty in exercising your rights, please contact the Data Protection Officer appointed by Inserm by e-mail (dpo@inserm.fr) or by post (Délégation à la Protection des Données, 101 rue de Tolbiac, 75013 Paris, France).

In addition, you have the right to lodge a complaint with the Commission Nationale de l'Informatique et des Libertés - CNIL (French data protection authority) via the following link **https://www.cnil.fr/fr/cnil-direct/question/adresser-une-reclamation-plainte-la-cnil-quelles-conditions-et-comment** or CNIL - Service des Plaintes - 3 Place de Fontenoy - TSA 80715 - 75334 Paris CEDEX 07, France.

6) Storage life

Your data will be kept for up to 5 years for research purposes, for the time needed to analyze and publish the results, and then archived for 15 years or more according to national requirements.

11. STUDY APPROVAL

This study received an initial favorable opinion from the ethics committee <name of EC> on xx/xx/xxxx and initial authorization from Regulatory Agency on xx/xx/xxxx.

12. INSURANCE

In accordance with the provisions of the French Public Health Code, the sponsor Inserm-ANRS MIE assumes the harmful consequences of the study for you and your beneficiaries.

To this end, Inserm-ANRS MIE has taken out an insurance policy with <insurer name, address and policy number> covering its civil liability.

13. CONTACT

If you encounter any problems or have any questions in connection with your participation in this study, before or during your treatment, you can contact your doctor using the information below:

Doctor Name :	*[site investigator name]*

Adress :	*[Site adress]*
Phone number :	*[Investigator phone number]*

MAIN STUDY

Version n°2.0 of 21/05/2025 approved by ethics committee <name of EC> on xx/xx/xxxx

Sponsor: Inserm-ANRS MIE **Coordinating investigators: Pr Beatriz GRINSZTEJN & Dr Pierre SELLIER**

Mrs, Mr .... *(Full name)*

I hereby certify:

- To have received the information letter version n°2.0 of 21/05/2025,
- To have had the opportunity to ask all the questions I wished about the nature, objectives, potential risks and constraints of my participation in this study,
- To have had sufficient time for reflection between information and consent.

I understood the constraints, in particular the regular hospital visits and blood sampling, and the benefits of taking part in this 96-week study.

I understand that I am free to interrupt my participation in this study at any time without having to explain why, but I will do my best to inform the doctor treating me. This will not affect the quality of my future care. I have been assured that the decisions that will be necessary for my health will be made at all times in accordance with the state of knowledge about HIV-1 infection in antiretroviral treatment-naive individuals.

I have noted that blood samples will be taken during the study and stored pseudonymized. They will be used to perform the analyses required for the study.

I accept that the data recorded during this study will be collected, processed and computerized. I understand that the right of access provided by the amended French Act of 6 January 1978 on Data Processing, Data Files and Individual Liberties and the General Data Protection Regulation (GDPR - Regulation (EU) 2016/679) applies at all times to the study doctor who is monitoring me as part of the study and that I may exercise my right of rectification and opposition.

My consent in no way relieves the study organizers of their responsibilities. I retain all rights guaranteed by law.

At the end of the study, **I will be informed** of the overall results by the study doctor.

I have been informed that further use of my data will be made available on the ANRS MIE website (currently www.anrs.fr) in a section dedicated to clinical study participants.

I have been informed by the information letter that my blood samples may be used at the end of the study for other research on HIV infection and its associated pathologies, emerging infectious and cardio-metabolic diseases. This opposition to subsequent re-use, whether total or specific to a search, may be made at any time.

**I freely agree to participate in this study under the conditions specified in the information letter**. *YES must be ticked to participate in the study.*	☐ Yes ☐ No
**I accept that my samples collected and the data associated in the context of the study may be used for further research on HIV infection and its associated pathologies, emerging infectious and cardio-metabolic diseases;** *if you tick NO, the samples will be destroyed once the study objectives have been achieved and will not be used for further research. If you tick NO, your participation within the study will not be impacted.*	☐ Yes ☐ No
**I agree that the data collected in the course of the study may be used for further research on HIV infection and its associated pathologies, emerging infectious and cardio-metabolic diseases in a pseudonymized way;** *if you tick NO, your data will not be re-used in any further research. If you tick NO, your participation within the study will not be impacted.*	☐ Yes ☐ No

Date: __ __ ____	**Signature of participant (or thumbprint/mark if illiterate):**
**If the participant is unable to give written consent,** an impartial witness, by signing, certifies that the participant has received and understood the information about the study, has obtained answers to his/her questions and freely consents to his/her participation in the study **(***the field below for the impartial witness does not need to be completed if the participant is capable of giving written agreement and free and informed consent).*	
Full witness name:<br><br>Date: __ __ ____	**Signature of impartial witness:**
**I, Dr/Pr.., hereby certify that I have given the participant all the information concerning this study, answered any questions and obtained the participant's consent.**	
Date: __ __ ____<br><br>Service name:<br>Address:<br>Phone:	**Signature of physician:**
