# Supplementary material for: Doravirine versus dolutegravir-based regimen in antiretroviral treatment-naive people living with HIV-1 (ANRS0392s ELDORADO): protocol for an international, open-label, randomised, non-inferiority, phase III trial
Source: BMJ Open. 2026 Feb 5;16(2):e110560. doi: 10.1136/bmjopen-2025-110560 (PMC12878352; doi:10.1136/bmjopen-2025-110560)
Supplement: Supplementary file 2 [file bmjopen-2025-110560.reviewer_comments.pdf]

PEER REVIEW HISTORY

BMJ Open publishes all reviews undertaken for accepted manuscripts. Reviewers are asked to complete a checklist review form and are provided with free text boxes to elaborate on their assessment. These free text comments are reproduced below.

ARTICLE DETAILS

Title (Provisional)

Doravirine versus dolutegravir-based regimen in antiretroviral treatment-naive people living with HIV-1 (ANRS0392s ELDORADO): protocol for an international, open-label, randomized, non-inferiority, phase III trial

Authors

L'hostellier, Anthony; Kouanfack, Charles; Chazallon, Corine; Wagner-Cardoso, Sandra; Eholie, Serge Paul; Banze, Neiva; Halue, Guttiga; Capeau, Jacqueline; Delaugerre, Constance; MOH, Raoul; Bonnet, Fabrice; Mfeukeu Kuate, Liliane; Jaquet, Antoine; Perazzo, Hugo; Bernard, Charlotte; Bastard, Jean-Philippe; Goldwirt, Lauriane; Vilquin, Paul; Nhassengo, Pedroso Pedro; Lavalée, Margot; Minvielle, Nicolas; Dodd, PJ; Marcy, Olivier; Molina, Jean-Michel; Grinsztejn, Beatriz; Sellier , Pierre O.

VERSION 1 - REVIEW

**Reviewer**	**1**
**Name**	**Francis, Joel**
**Affiliation**	**University of the Witwatersrand Johannesburg Faculty of Health Sciences, Family Medicine and Primary Care**
**Date**	**18-Dec-2025**
**COI**	**None**

It is a timely study, and the protocol is detailed and well-written. I only have a few minor comments below.

1. What is the rationale of using the cut-off of RNA copies of ≥ 1000? The current guidelines have a much more stringent viral load cut-off of ≥ 50.

2. In the abstract, there is a mention of recruiting GIV treatment naïve – it would be helpful to provide details on how this would be assessed.

3. It is indicated that the randomisation will be stratified by screening HIV-1 RNA load (≤100,000 or >100,000 copies/mL) and by country – what is the rationale for this?

VERSION 1 - AUTHOR RESPONSE

Reviewer: 1
Prof. Joel Francis, University of the Witwatersrand Johannesburg Faculty of Health Sciences
Comments to the Author:
It is a timely study, and the protocol is detailed and well-written. I only have a few minor comments below.

1. What is the rationale of using the cut-off of RNA copies of ≥ 1000? The current guidelines have a much more stringent viral load cut-off of ≥ 50.

A rational of using the cut-off has been added as in the "Study population and recruitment" section: "The rationales of using the cut-off of baseline RNA levels of ≥ 1000 copies/mL were: i) this cut-off was previously used in the two large RCT comparing doravirine to ritonavir-boosted darunavir (11), and to EFV (24), and in NAMSAL trial, comparing DTG to EFV (6). In ADVANCE trial, the cut-off was 500 copies/mL ii) sites participating to this international trial used the cut-off 1000 copies/mL in routine."

2. In the abstract, there is a mention of recruiting GIV treatment naïve – it would be helpful to provide details on how this would be assessed.

Initial HIV-1 treatment-naïve status will be assessed in accordance with the national guidelines in each participating country. HIV diagnosis will follow the standard diagnostic algorithm, including an initial rapid diagnostic test (RDT) followed by a discriminatory confirmatory assay performed during screening visit to exclude HIV-2 infection or HIV-1/HIV-2 co-infection. Treatment-naïve status will be determined based on a detailed clinical history, review of available medical records, and confirmation of no prior exposure to antiretroviral therapy. This approach reflects routine clinical practice in the study settings and ensures reliable identification of ART-naïve participants.

This justification has been added in the article, section Study population and recruitment

3. It is indicated that the randomization will be stratified by screening HIV-1 RNA load (≤100,000 or >100,000 copies/mL) and by country – what is the rationale for this?

Randomization will be stratified by baseline HIV-1 RNA level (≤100,000 vs >100,000 copies/mL) because baseline viral load is a well-established prognostic factor for virological response to antiretroviral therapy. Higher baseline HIV-1 RNA levels have been associated with delayed viral suppression and an increased risk of early virological failure. The threshold of 100,000 copies/mL is widely used in clinical trials and international guidelines as a clinically meaningful cut-off to distinguish patient populations with differing virological risk profiles. Stratifying randomization by this variable ensures comparability between treatment arms, minimizes potential confounding, and strengthens the internal validity of the study outcomes. Moreover, randomization stratified by baseline HIV-1 RNA level (≤100,000 vs >100,000 copies/mL) has been used previously in the two large RCT comparing doravirine to ritonavir-boosted darunavir (10), and to EFV (24), as in NAMSAL and ADVANCE trials, comparing DTG to EFV (6, 28). Randomization will also be stratified by country as data concerning virological success are scares, mainly for doravirine, towards non-B HIV-1 strains; moreover, weight gain in PLHIV taking DTG has been described in sub-Saharan people and can differ in participating countries.

This justification has been added in the article, section Randomization

*** ***

COI statement(s):

Reviewer: 1
If you have selected ‘Yes’ above, please provide details of any competing interests.: Not applicable.
